# Estimated Exposure to Arsenic in Breastfed and Formula-Fed Infants in a United States Cohort

**DOI:** 10.1289/ehp.1408789

**Published:** 2015-02-23

**Authors:** Courtney C. Carignan, Kathryn L. Cottingham, Brian P. Jackson, Shohreh F. Farzan, A. Jay Gandolfi, Tracy Punshon, Carol L. Folt, Margaret R. Karagas

**Affiliations:** 1 Children’s Environmental Health and Disease Prevention Research Center at Dartmouth, Hanover, New Hampshire, USA; 2 Department of Biological Sciences, and; 3 Trace Element Analysis Laboratory, Department of Earth Sciences, Dartmouth College, Hanover, New Hampshire, USA; 4 Department of Epidemiology and Norris Cotton Cancer Center, Geisel School of Medicine at Dartmouth, Hanover, New Hampshire, USA; 5 Department of Pharmacology and Toxicology, University of Arizona, Tuscon, Arizona, USA; * These authors contributed equally to this work.

## Abstract

Background: Previous studies indicate that concentrations of arsenic in breast milk are relatively low even in areas with high drinking-water arsenic. However, it is uncertain whether breastfeeding leads to reduced infant exposure to arsenic in regions with lower arsenic concentrations.

Objective: We estimated the relative contributions of breast milk and formula to arsenic exposure during early infancy in a U.S. population.

Methods: We measured arsenic in home tap water (*n* = 874), urine from 6-week-old infants (*n* = 72), and breast milk from mothers (*n* = 9) enrolled in the New Hampshire Birth Cohort Study (NHBCS) using inductively coupled plasma mass spectrometry. Using data from a 3-day food diary, we compared urinary arsenic across infant feeding types and developed predictive exposure models to estimate daily arsenic intake from breast milk and formula.

Results: Urinary arsenic concentrations were generally low (median, 0.17 μg/L; maximum, 2.9 μg/L) but 7.5 times higher for infants fed exclusively with formula than for infants fed exclusively with breast milk (β = 2.02; 95% CI: 1.21, 2.83; *p* < 0.0001, adjusted for specific gravity). Similarly, the median estimated daily arsenic intake by NHBCS infants was 5.5 times higher for formula-fed infants (0.22 μg/kg/day) than for breastfed infants (0.04 μg/kg/day). Given median arsenic concentrations measured in NHBCS tap water and previously published for formula powder, formula powder was estimated to account for ~ 70% of median exposure among formula-fed NHBCS infants.

Conclusions: Our findings suggest that breastfed infants have lower arsenic exposure than formula-fed infants, and that both formula powder and drinking water can be sources of exposure for U.S. infants.

Citation: Carignan CC, Cottingham KL, Jackson BP, Farzan SF, Gandolfi AJ, Punshon T, Folt CL, Karagas MR. 2015. Estimated exposure to arsenic in breastfed and formula-fed infants in a United States cohort. Environ Health Perspect 123:500–506; http://dx.doi.org/10.1289/ehp.1408789

## Introduction

Arsenic occurs naturally in bedrock and is a common global contaminant of well water (Meharg 2005). It is a known human carcinogen associated with skin, lung, bladder, kidney, and liver cancer and can also affect neurological, respiratory, cardiovascular, immunological, and endocrine systems [International Agency for Research on Cancer 2004; National Research Council (NRC) 1999, 2014; Naujokas et al. 2013; Tseng 2009]. The U.S. Environmental Protection Agency (EPA) has set a maximum contaminant level (MCL) of 10 μg/L for public drinking water (U.S. EPA 2001). Private well water, however, is not subject to regulation and is the primary water source in many rural parts of the United States. In New Hampshire, these wells serve approximately 40% of the population, with approximately 10% of wells containing arsenic concentrations exceeding the MCL (Nuckols et al. 2011; Peters et al. 2006).

Early life is a period of heightened vulnerability to arsenic exposure (Farzan et al. 2013a; Tseng 2009; Vahter 2008). In populations where drinking-water arsenic concentrations are high, early-life exposure has been associated with increased fetal mortality, decreased birth weight, and diminished cognitive function (NRC 2014). Children in these highly exposed populations have different arsenic excretion rates and metabolic profiles than adults, suggesting that children may be more sensitive to arsenic toxicity (Concha et al. 1998; Fängström et al. 2009). Moreover, effects of chronic early-life exposure can continue into adulthood, as suggested by increased occurrences and/or severity of lung disease, cardiovascular disease, and cancer later in life (Naujokas et al. 2013; Smith et al. 2006). Much less is known about the consequences of low-level exposure, particularly in early life. However, *in utero* exposure to low levels of arsenic has been associated with increased infant infections and the severity of infections in U.S. infants (Farzan et al. 2013b) and childhood exposure with decreased IQ (Wasserman et al. 2014).

Infants and children often experience higher total contaminant exposures than adults because their intakes adjusted for body mass are relatively high (Tsuji et al. 2007) and dietary diversity is low [European Food Safety Authority (EFSA) 2009]. Newborn infants have a limited diet, ingesting breast milk or formula almost exclusively for the first 4–6 months of life. Recent studies suggest that formula powder can contain low concentrations of arsenic (Food and Drug Administration 2013; Jackson et al. 2012; Ljung et al. 2011; Sorbo et al. 2014). This suggests that both components of reconstituted formula—the powder and the water with which it is mixed—can be sources of arsenic exposure for formula-fed infants. Conversely, breast milk has been found to have relatively low concentrations of arsenic (Björklund et al. 2012), even in women with high exposure via their drinking water (e.g., Concha et al. 1998; Fängström et al. 2008; Samanta et al. 2007).

We therefore hypothesized that breastfed infants in New Hampshire have lower exposure to arsenic compared with formula-fed infants. We tested this hypothesis by measuring urinary arsenic concentrations in a subset of infants enrolled in the New Hampshire Birth Cohort Study (NHBCS). In addition, we used a modeling approach to estimate daily intake of arsenic from breast milk and formula for the larger cohort of NHBCS infants, as well as infants consuming formula made with tap water containing arsenic concentrations of potential toxicological and regulatory interest: 1 μg/L, a level considered to be relatively low (NRC 2014); 5 μg/L, the MCL in New Jersey (New Jersey Administrative Code 7:10 2011); and 10 μg/L, the current U.S. EPA MCL (U.S. EPA 2001).

## Materials and Methods

*The NHBCS*. In January 2009, we began recruiting pregnant women ranging in age from 18 to 45 years who were receiving prenatal care at study clinics in New Hampshire (Farzan et al. 2013b; Gilbert-Diamond et al. 2011). Enrollment criteria included the use of a private, unregulated well at the home occupied since their last menstrual period and plans to stay in the current residence through delivery. This study was reviewed and approved by the Committee for the Protection of Human Subjects (CPHS) at Dartmouth College, and all participants in the study provided written informed consent in accordance with CPHS guidelines.

*Maternal questionnaire*. Participants were asked to complete a prenatal medical history and lifestyle questionnaire that included questions about sociodemographic factors, health history, personal habits, home water source, use of water filters, and home water consumption. Characteristics of the study population are described in Table 1.

**Table 1:** Selected characteristics of mothers and infants in the feeding study subset (*n *= 72) and the larger New Hampshire Birth Cohort Study (*n *= 937).

Characteristic	Feeding study subset^*a*^ [mean (range) or % (*n*)]	Larger NHBCS^*b*^ [mean (range) or % (*n*)]
Maternal characteristics
Age at enrollment (years)	32 (22–43)	31 (19–45)
< 20	0 (0)	1 (16)
20–29	29 (21)	31 (292)
30–35	40 (29)	46 (427)
> 35	31 (22)	21 (200)
Education
< 11th grade	1 (1)	1 (9)
High school graduate or GED	7 (5)	10 (90)
Junior college, some college, technical school	19 (13)	21 (182)
College graduate	36 (25)	40 (341)
Postgraduate schooling	37 (26)	28 (238)
Relationship status
Single	4 (3)	10 (88)
Married	93 (65)	87 (745)
Separated or divorced	3 (2)	3 (27)
Smoked during pregnancy
Yes	7 (5)	6 (53)
No	93 (65)	94 (821)
Infant characteristics
Sex
Male	54 (38)	49 (438)
Female	46 (33)	51 (454)
Race
White	97 (62)	99.3 (841)
Other	3 (2)	0.7 (6)
Gestational age (weeks)		39.4 (27–45)
Tap-water As (µg/L)^c,^*	0.15 (< 0.01–29.4)	0.44 (< 0.01–189)
< 1	77 (54)	59 (518)
1–10	21 (15)	30 (261)
> 10	1 (1)	10 (95)
^***a***^NHBC participants who provided both an infant urine sample and 3-day food diary between 1 and 3 months of age. Sum of subjects may be less than the total sample size due to missing data: Two subjects were missing maternal age, education, relationship status, and smoking during pregnancy; one was missing infant sex; and eight were missing infant race. ^***b***^Sum of subjects may be less than the total sample size due to missing data: 77 subjects were missing maternal education and relationship status, 63 were missing smoking during pregnancy, 45 were missing infant sex, 91 were missing infant race, and 63 were missing tap-water arsenic. ^***c***^Median value: data are log-normally distributed. *Significantly different from the full NHBCS in the distribution among the three categories < 1, 1–10, and > 10 μg/L (χ^2^ = 9.53, *p* = 0.01, degrees of freedom = 2).		

*Home tap water*. On enrollment, participants were asked to provide water samples from the tap in their kitchen. They were given a commercially washed, mineral-free, high-density polyethylene collection bottle that meets U.S. EPA standards for water collection (I-Chem; Cole-Parmer). Bottles were kept in clean, sealed plastic bags, and participants were provided with specific instructions to minimize contamination. If filtered tap water was used for drinking, they were asked to provide a filtered sample. Each participant was given mailing materials to return the samples to the study office, where they were stored at –20°C or lower.

Water samples were analyzed by inductively coupled plasma mass spectrometry (ICP-MS) at the Trace Element Analysis (TEA) Core at Dartmouth using a quadrupole collision cell 7500c Octopole Reaction System (Agilent) and helium as a collision gas to remove polyatomic interferences. All sample preparations and analyses were carried out in a trace metal–clean HEPA filtered–air environment. Analytical blanks and potential instrumental drift were monitored, and instrument standardization and reproducibility were performed with National Institute of Standards and Technology [NIST-traceable standards, e.g., NIST Standard Reference Material (SRM) 1640a for water] and certified standard reference materials. Water samples were acidified with Optima HNO_3_ (Fisher Scientific) to 0.5% vol/vol after thawing and a 5-mL aliquot was taken for total arsenic analysis. The analytical uncertainty of these procedures is typically ± 3–5% with a weighted linear regression calibration method. The detection limit ranged from 0.009 to 0.074 μg/L (mean, 0.014 μg/L). All arsenic in home tap water was assumed to be inorganic based on previous studies (Meacher et al. 2002; NRC 1999).

*Infant urine and food diary*. A subset of NHBCS mothers who delivered their babies between July 2012 and April 2013 were asked to complete a 3-day food diary and collect a urine sample from their infant at approximately 6 weeks of age. For each feeding, the mother or caretaker recorded the time, type of food or drink item (e.g., infant formula, breast milk, or expressed breast milk), amount consumed (e.g., ounces of formula or expressed breast milk, minutes of breastfeeding), amount of water mixed with powdered formula (if any), and the source of that water (e.g., home tap, brand of bottled water).

Urine samples were collected on the third day of the food diary using provided diapers and cotton pads (Shiseido) and a protocol adapted from Fängström et al. (2008). If the pads were soiled by feces, mothers were instructed to discard the pads and make another attempt at collection. The saturated pads were placed in a collection cup, sealed in a polyethylene bag, stored in a cooler with frozen ice packs, and brought to the mother’s 6-week postpartum appointment later that day. Samples were stored upright at 4°C and couriered to the Pathology Department at Dartmouth Hitchcock Medical Center (DHMC) where, within approximately 24 hr, the urine was squeezed from the cotton pads, divided into aliquots, and frozen at –80°C. Specific gravity was measured using a handheld refractometer with automatic temperature compensation (ATAGO® PAL-10S; Atago U.S.A., Inc.). No more than 36 hr elapsed between sample collection and storage at –80°C. All urine samples were analyzed in a single batch within 1 year of collection. While developing this sampling method, we tested 11 commercially available cotton pads. Blanks produced using the selected brand (Shiseido) had the lowest mean (± SE) concentrations of arsenic (0.021 ± 0.007 μg/L) relative to the other products tested (means of 0.03–0.58 μg/L).

Urine samples were analyzed for total arsenic and individual arsenic species at the Arizona Laboratory for Emerging Contaminants at the University of Arizona. Urine samples were thawed, vortexed, filtered at 0.45 μm, and diluted 10-fold in 10 mM ammonium carbonate, 5 mM EDTA. These diluted samples were analyzed for total urinary arsenic by ICP-MS with an external calibration prepared in a synthetic urine matrix using germanium as an internal standard. The limit of detection (LOD) for total urinary arsenic was 0.05 μg/L.

The arsenic speciation method quantitatively determines levels of inorganic arsenic (As^III^ and As^V^), monomethylarsonic acid (MMA), dimethylarsinic acid (DMA), arsenobetaine (AsB), and arsenocholine. All speciation analyses were performed using a high-performance liquid chromatography (HPLC) Dionex GP50 pump and a Hamilton PRP100X column connected to a collision cell ICP-MS (Larsen et al. 1993; Le et al. 2000; Wei et al. 2001). The mobile phase was a gradient of 10–50 mM ammonium carbonate at pH 8.5 over 13 min. The sample volume was 100 μL and the column flow rate was 1 mL/min. The separated arsenic was detected by ICP-MS using time-resolved analysis at *m/z* 75. We included eight duplicate and three composite control samples for quality control purposes.

*Breast milk*. We analyzed arsenic concentrations in breast milk from a subsample of nine NHBCS mothers who had indicated intent to breastfeed on a prenatal questionnaire and who delivered their infants between July 2012 and March 2013. Samples were collected at infant’s age 2–7 weeks by each mother at home into her own breast milk storage bag or bottle, stored at 4° or –20°C, and delivered to DHMC. Once received, all samples were frozen at –80°C.

One milliliter of breast milk was weighed into a Teflon digestion vessel, and 1 mL of 9:1 HNO_3_:HCl (Optima, Fisher Scientific) was added to the vessel. The vessels were capped and microwave digested (MARS6; CEM Corporation) at 200°C for 20 min, and then diluted to approximately 10 mL. Digested samples were analyzed by ICP-MS (Agilent 7700x; Agilent Technologies) at the Dartmouth TEA Core as described above. Initial and continuing calibration checks followed procedures outlined by the U.S. EPA (2007). Duplicate samples, blank digests (*n* = 4), and NIST SRM 1849a were included for quality control. Although NIST SRM 1849a is not certified for arsenic, we assessed recovery for the certified element selenium, which is prone to the same severe matrix effects as arsenic in these complex milk matrixes. Recovery for selenium in NIST SRM 1849a was 105%. We measured arsenic in this SRM to be 4 ng/g, near the method detection limit. Average sample spike recovery for As (*n* = 2) was 112% and relative percent difference of duplicate analysis (*n* = 2) was 13%. Arsenic recovery of the fortified blank (taken through the acid digestion process) was 98%.

*Data analysis*. We used chi-square and *t*-tests to evaluate differences in demographic characteristics and natural log (ln)–transformed household tap water arsenic between the full NHBC and feeding study subset.

Using the 2 full days of the 3-day diaries (days 1 and 2), we calculated the average number of feedings of both breast milk and formula per day, number of minutes spent breastfeeding, amount of formula and expressed breast milk consumed, and ounces of water consumed in reconstituted formula. Given these data, we assigned infants to one of three feeding categories: exclusively breastfed, exclusively formula-fed, or mixed (consumed both breast milk and formula). Exclusive breastfeeding included meals of expressed (pumped) breast milk.

Our statistical models focused on urinary arsenic (UAs) measured as total arsenic minus arsenobetaine. Arsenobetaine was excluded because it is thought to be nontoxic and to pass through the body without being metabolized (Tseng 2009); no arsenocholine was detected. UAs values below the LOD for total arsenic were assigned a uniform random variate between zero and the LOD (Helsel 1990), and the arsenobetaine concentration was assumed to be zero if it was not detected. We then used parametric general linear models (PROC GLM in SAS; SAS Institute Inc.) to assess the association of UAs with potential predictors of exposure, after adjusting for specific gravity to account for urinary dilution (Nermell et al. 2008) by including it as a covariate in the model. Potential predictors evaluated included feeding mode, ounces per day of formula, minutes per day breastfed, home tap water arsenic concentration, infant age at urine collection, infant sex, maternal education, maternal cigarette smoking, and maternal secondhand smoke exposure. Because the maternal and infant demographic variables were not associated with infant UAs at α = 0.05, they were not included as covariates in our final models. We also evaluated whether the probability of detecting each arsenic species differed between exclusively breastfed and exclusively formula-fed infants using Fisher’s exact test. All statistical analyses were performed using SAS version 9.3, with α = 0.05 as the level of statistical significance.

*Exposure models*. We used a deterministic modeling approach to evaluate potential arsenic exposure (as daily arsenic intake per kilogram of body mass) for infants who were exclusively breastfed versus exclusively formula-fed, both for the full NHBCS and for broader populations of infants consuming tap water at specified concentrations. For exclusively formula-fed infants, we estimated arsenic exposure from powdered formula reconstituted with bottled water, NHBCS household tap water, and water containing arsenic at three concentrations of toxicological and regulatory interest: 1, 5, and 10 μg/L.

In each of our models, we multiplied a standardized ingestion rate (IR; liters per day) by the concentration of arsenic (C; micrograms per liter) in the respective exposure medium (breast milk, formula powder, or water):

Estimated exposure, μg/day = IR × C. [1]

The standardized ingestion rate, used for all infants, was calculated from the food diaries of our exclusively formula-fed infants (mean, 0.81 L/day; range, 0.58–1.18 L/day). For arsenic concentration inputs (C), we used minimum, median, and maximum values measured in this study for breast milk (*n* = 9, Table 2) and home tap water arsenic (*n* = 874, Table 2) and from published data for formula powder and bottled water. Data on arsenic in formula powder came from a New Hampshire market basket study of 15 popular formula powders from 5 name brands, analyzed in triplicate (Jackson et al. 2012): median, 1.1 μg/L; and range, 0.3–1.8 μg/L. Data on arsenic in bottled water came from a market basket study of bottled water sold in California (Sullivan and Leavey 2011): median, 0.62 μg/L; and range, 0.07–1.93 μg/L. Because body weight data are not yet available for our study population, we standardized exposures to body weight (BW; kilograms) using data on 1*-* to 3-month-old infants from the U.S. EPA Child-Specific Exposure Factors Handbook (mean, 5.6 kg; range, 4.5–7.3 kg) (U.S. EPA 2008):

**Table 2:** Summary statistics for measured arsenic concentrations (µg/L) in samples of household tap water, infant urine, and maternal breast milk collected as part of this study.

Matrix	*n*	LOD	Percent detected (*n*)	Minimum	Percentile			Maximum
					25th	50th	75th	
Home tap water		0.01						
Full cohort	874		84 (736)	< LOD	0.07	0.44	2.72	189
Substudy	70		71 (50)	< LOD	0.03	0.15	0.57	29
Infant urine^*a*^	72	0.05	97 (70)	< LOD	0.07	0.17	0.37	2.9
Breast milk^*b*^	9	0.22	67 (6)	< LOD	0.25	0.31	0.44	0.62
^***a***^Urinary arsenic defined as total arsenic minus arsenobetaine; data shown here were not adjusted for specific gravity. Detection frequency is given for total arsenic. ^***b***^Assuming 1:1 conversion from ng/g to μg/L, following Björklund et al. (2012).								

Estimated exposure, μg/kg/day = (IR × C) ÷ BW. [2]

For each model, we estimated exposure using minimum, median, or maximum parameter inputs. Median estimated exposure was calculated using mean ingestion rate, mean body weight, and the median concentration of arsenic. Minimum and maximum inputs were paired by ingestion rate and body weight because of their underlying correlation.

Minimum estimated exposure, μg/kg/day = (_min_IR × _min_C) ÷ _min_BW. [3]

Median estimated exposure, μg/kg/day = (_mean_IR × _median_C) ÷ _mean_BW. [4]

Maximum estimated exposure, μg/kg/day = (_max_IR × _max_C) ÷ _max_BW. [5]

## Results

*Characteristics of participants in the infant feeding substudy*. As of 1 September 2013, a total of 1,036 mother–infant pairs were enrolled in the NHBCS; 937 had demographic data and 874 had data on their home tap-water arsenic concentration available for exposure modeling.

During the recruitment period for the infant feeding substudy (July 2012—April 2013), we mailed study collection packets to 136 NHBCS participants whose infants had reached 6 weeks of age. A total of 97 (70%) samples were returned by June 2013. Of these, 84 (87%) had a completed 3-day diary and 82 (85%) had sufficient urinary volume for arsenic analysis, resulting in a subsample of 72.

Demographic characteristics of this subset were similar to the larger cohort (Table 1). The mean (± SD) maternal age was 32.3 ± 5.2 years at the time of enrollment, and most mothers were college graduates (36%) or had attended some postgraduate schooling (37%). Most of the mothers were married (93%) and reported that they did not smoke and were not exposed to secondhand smoke during pregnancy (93%). Slightly less than half of the infants were female (46%), and 97% were white.

*Infant feeding substudy*. Using all of the food diaries collected at approximately 6 weeks of age (*n* = 115), 70% of mothers reported that their infants received exclusively breast milk, 13% received exclusively formula, and 17% received a combination of breast milk and formula (see Supplemental Material, Table S1). Breastfed infants had a mean (± SD) of 9.5 ± 2.6 feedings across 142 ± 86.9 min per day, whereas formula-fed infants consumed an average volume of 0.81 ± 0.15 L/day across 8.0 ± 1.8 feedings. For infants receiving both breast milk and formula, the average reported volume of formula consumed was 0.30 ± 0.24 L/day across 3.8 ± 2.8 feedings. Among the infants who received any formula, 70% of mothers reported using their home tap water to prepare formula more than half the time, whereas the other 30% reported using bottled water more than half the time.

We detected a range of concentrations of arsenic in home tap water (*n* = 874 for the full study; *n* = 70 for the substudy) and infant urine (*n* = 72) (Table 2). Across the whole cohort, median arsenic concentrations in tap water were relatively low (< 1 μg/L). Overall, about 10% of NHBCS families had tap water that exceeded the MCL of 10 μg/L, with a maximum concentration of 189 μg/L (Table 1). In the infant substudy (*n* = 72), significantly fewer homes (1%) had tap water above the MCL (Table 1), and the maximum observed concentration was much lower (29 μg/L) (Table 2). Nevertheless, we detected total arsenic in 97% of our infant urine samples. Detection frequencies for AsB, inorganic arsenic, MMA, and DMA were 19%, 24%, 8%, and 38%, respectively, likely due to a combination of low total arsenic concentrations and relatively high detection limits for the individual species (see Supplemental Material, Table S2). Mean (± SD) specific gravity of these samples was 1.003 ± 0.0014.

**Figure 1:**
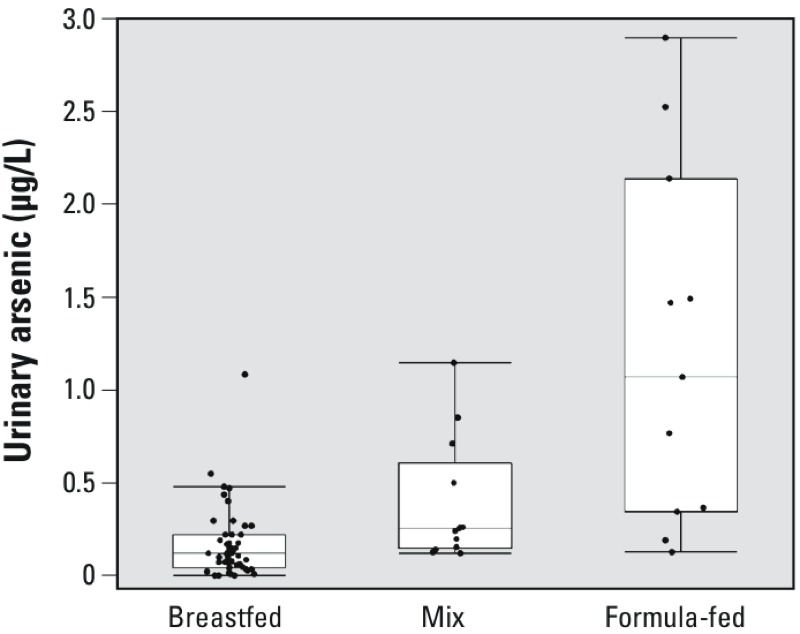
Urinary arsenic (total arsenic minus arsenobetaine, not adjusted for specific gravity) in approximately 6‑week‑old infants by predominant feeding mode: exclusively breastfed (*n *= 48), mixed (*n *= 13), and exclusively formula-fed (*n *= 11). Boxes represent quartiles, the lines within the boxes represent the median, and each whisker represents the quartile ± 1.5 times the interquartile range. Dots represent individual results.

Urinary arsenic concentrations (UAs, as total arsenic minus arsenobetaine) were lowest in exclusively breastfed infants and highest in exclusively formula-fed infants; infants fed both formula and breast milk had intermediate concentrations of UAs (Figure 1). In a general linear regression model controlling for urinary specific gravity [β = 222; 95% confidence interval (CI): 19.8, 425; *p* = 0.03 for the difference in ln-UAs with a 1-unit increase in specific gravity], the geometric mean UAs concentration for exclusively formula-fed infants was 7.5 times higher than for breastfed infants (β = 2.02; 95% CI: 1.21, 2.83; *p* < 0.0001); concentrations in infants fed both breast milk and formula were three times higher (β = 1.08; 95% CI: 0.34, 1.83; *p* = 0.005). The 2.5-fold difference between exclusively formula-fed infants and infants fed both breast milk and formula was marginally significant (β = 0.94; 95% CI: –0.04, 1.91; *p* = 0.06).

Ingestion rates of formula and breast milk were also significant predictors of UAs. Among infants who were not exclusively breastfed, a 1-oz increase in daily formula consumption was associated with a 2.6% increase in UAs (β = 0.026; 95% CI: 0.010, 0.042; *p* = 0.003), whereas minutes spent breastfeeding per day was inversely associated (β = –0.004; 95% CI: –0.006, –0.002; *p* = 0.001). We observed no association between minutes of breastfeeding per day and ln-UAs (β = –0.001; 95% CI: –0.003, 0.001; *p* = 0.43) among exclusively breastfed infants. There was a suggestion of a positive association between ln-UAs and the ln-transformed concentration of arsenic in home tap water among infants who were not exclusively breastfed (β = 0.16; 95% CI: –0.12, 0.44; *p* = 0.24; *n* = 22), with limited statistical power and low exposure levels (only one water sample > 1 µg/L, 8.6 μg/L). No association with water arsenic was observed among exclusively breastfed infants (β = 0.008; 95% CI: –0.17, 0.19; *p* = 0.93; *n* = 48).

With respect to individual arsenic species (see Supplemental Material, Table S2), DMA and MMA were detected more frequently among exclusively formula-fed than among exclusively breastfed infants (Fisher’s exact test, *p* < 0.0001 and *p* = 0.005, respectively). DMA was detected in urine from all 11 formula-fed infants compared with only 9 of 48 of breastfed infants; MMA was not detected in any of the 48 breastfed infants, but was detected in 3 of 11 formula-fed infants. Detection frequencies for inorganic arsenic and AsB did not vary with feeding mode (both *p* > 0.4).

*Breast milk*. Arsenic concentrations in breast milk samples were low (median, 0.31 μg/L; maximum, 0.62 μg/L), with detectable arsenic in only five of the nine samples (Table 2). Because of these low concentrations, we did not perform arsenic speciation analyses. The median tap-water arsenic concentration for the nine mothers who provided breast milk was 0.26 μg/L (range, < 0.01–8.9 μg/L). Six of the nine mothers also participated in the feeding substudy.

**Figure 2:**
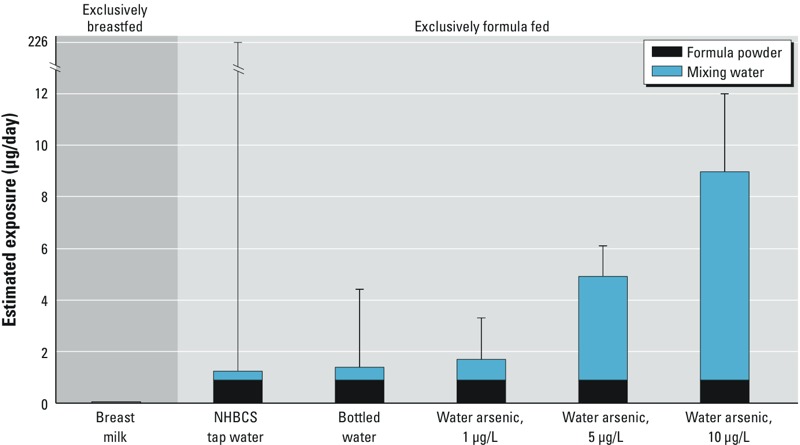
Estimated arsenic exposure for exclusively breastfed and exclusively formula-fed infants at 1–3 months of age, based on our exposure models and different potential water sources for reconstituting formula powder. Estimates for breastfed babies were based on a subsample of infants from the NHBC (Table 2; *n *= 9). Estimates for formula-fed babies were based on the median and maximum concentration of arsenic in formula powder measured as part of a previous market basket study for the study population (Jackson et al. 2012) and either measured home tap-water arsenic concentrations (Table 2, *n *= 874) or previously measured median and maximum concentrations of arsenic in bottled water from California (Sullivan and Leavey 2011). Bar heights indicate median estimated exposure, and error bars indicate maximum estimated exposure.

*Exposure models*. Using median tap-water arsenic from the larger NHBCS in our exposure model, we observed that estimated median arsenic exposure was 5.5 times higher for exclusively formula-fed infants (0.22 μg/kg/day) compared with exclusively breastfed (0.04 μg/kg/day) infants (Figure 2; see also Supplemental Material, Table S3). Because measured median tap-water arsenic was low (0.44 μg/L), exposure via formula powder [0.16 μg/kg/day, estimated from measured tap-water arsenic plus the median arsenic concentration in the 15 formulas measured by Jackson et al. (2012)] accounted for the majority (71%) of median estimated exposure. However, the maximum estimated exposure (31 μg/kg/day; Figure 2) was attributable almost entirely to the high tap-water arsenic concentration in a single household (189 μg/L). Median estimated exposures for formula mixed using bottled water were similar to using NHBCS tap water (0.25 vs. 0.22 μg/kg/day, respectively), but maximum estimated exposure was considerably lower with bottled water (just 0.60 μg/kg/day) due to the absence of outliers in the measurements of bottled water arsenic made by Sullivan and Leavey (2011) (Figure 2). Finally, the proportion of estimated arsenic exposure due to formula powder decreased with increasing tap-water concentrations, with 52% of exposure attributable to formula powder for water at 1 μg/L of arsenic compared with 10% for water at 10 μg/L (Figure 2).

## Discussion

In our study, infants who were fed exclusively with breast milk had lower exposures to arsenic than those fed exclusively with formula or a mix of formula and breast milk, as determined by both urinary biomarkers and exposure modeling. Moreover, urinary arsenic increased with formula consumption and decreased with minutes of breastfeeding among infants who were not exclusively breastfed. These findings are consistent with the lower median concentration of arsenic measured in breast milk (0.31 μg/L) in the NHBCS compared with the combined concentrations of arsenic in formula powder (median 1.1 μg/L) and tap water (median 0.44 μg/L).

Our finding of low arsenic in breast milk was consistent with a previous study in Uppsala, Sweden, where drinking-water arsenic is low (Björklund et al. 2012). Although our estimate should be interpreted with caution given the small sample size (*n* = 9), studies consistently document much lower arsenic in breast milk than in drinking water (Table 3). Together, these studies suggest that breastfeeding is likely to result in lower infant exposures to arsenic regardless of arsenic concentrations in drinking water. This finding is consistent with studies of other metals such as lead (Ettinger et al. 2004; Gulson et al. 2001) and demonstrates an important public health benefit of breastfeeding.

**Table 3:** Summary of reported arsenic concentrations (µg/L) in breast milk and drinking water.

Reference	Country	*n*	Sampling year(s)	Weeks postpartum	Breast milk		Drinking water	
					Median	Range	Median	Range
Grandjean et al. 1995	Faroe Islands	23	NA	< 1	1.6	0.1–4.4	NA	NA
Concha et al. 1998	Argentina	10	1995	< 1–28	2.3	0.85–7.7	190	157–219
Sternowsky et al. 2002	Germany	36	NA	< 1–13	< 0.3	< 0.3–2.8	NA	NA
Samanta et al. 2007	India	226	1996–2006	NA	17	< 2.0–49	140	10–1,380
Fängström et al. 2008	Bangladesh	79	2002–2003	8–12	1.0	0.25–19	78^*a*^	1–410
Björklund et al. 2012	Sweden	60	2000–2009	2–3	0.33	0.04–4.6	NA	NA
Sakamoto et al. 2012	Japan	9	NA	~ 12	1.4	0.4–1.80	NA	NA
Current study	USA	9	2012–2013	1.7–7	0.31	< 0.22–0.62	0.26^*b*^	< 0.01–8.9
NA, not available. ^***a***^As reported by Vahter et al. (2006) for a broader sample of the population in Matlab, Bangladesh (*n *= 2,330). ^***b***^Calculated from tap water data for the nine breast milk samples.								

Our exposure models further suggest that formula powder can make a large contribution to arsenic exposure for formula-fed infants when the arsenic concentration in drinking water is low (< 1 μg/L). Specifically, formula powder accounted for 71% of median estimated exposure in the NHBCS, suggesting that the powdered component of formula, rather than the mixing water, may be the primary source of exposure for many of the formula-fed infants in this population. This finding may be of particular concern given that the predominant form of arsenic in formula powder appears to be the more toxic inorganic species (Jackson et al. 2012). Identifying the sources of arsenic in formula powder could help reduce exposure for formula-fed infants if alternatives are available in the production process, consistent with earlier calls for greater attention to contaminants in infant formula (e.g., Ljung et al. 2011).

Median body weight–adjusted estimated arsenic exposures for NHBCS infants, assuming exclusive breast- and formula-feeding (0.04 and 0.22 μg/kg/day, respectively), were slightly lower than recent central-tendency estimates for European infants (mean, 0.24–0.43 μg/kg/day; EFSA 2014) and did not exceed the provisional tolerable weekly intake (PTWI) for arsenic previously used by the World Health Organization (15 μg/kg/week, or 2.1 μg/kg/day). Importantly, however, the WHO PTWI was withdrawn in 2010 as being insufficient to protect health, and recommendations have been made to reduce arsenic ingestion especially in young children (EFSA 2009, 2014). Therefore, our results reinforce recommendations for families with private wells to test for arsenic in their tap water and seek remediation if necessary.

Urinary arsenic concentrations in our U.S. infant population were lower than what has been observed in a highly exposed population in Bangladesh, even among breastfed children (Fängström et al. 2008). Mean urinary specific gravity in our infant subsample (1.003) was identical to that of 3-month-old Bangladeshi infants (Fängström et al. 2008), but lower than that of 18-month-old toddlers (1.009) (Hamadani et al. 2010). After adjustment to the average specific gravity, the median concentration of urinary arsenic in infant urine from the NHBCS (0.18 μg/L) was 6.5 times lower than 3-month-old Bangladeshi infants (1.2 μg/L) (Fängström et al. 2008). To our knowledge, no other study has investigated urinary arsenic in infants at 6 weeks of age from any region of the world, or breast milk arsenic in a U.S. population.

Limitations of our study include the lack of individual-level data for making individualized exposure estimates, the relatively small number of formula-fed infants, and the procedures for collecting breast milk. We were unable to calculate individualized exposure estimates for four reasons. First, we lacked body weight data. Second, we lacked data on ounces of milk ingested during breastfeeding events. Mothers reported the number of minutes on the breast, but we determined that extrapolation of minutes into ounces would be problematic due to the large variation in milk output both between and within individuals. Third, estimates of breast milk arsenic were available for only nine infants, not all of whom participated in our feeding substudy. Finally, although we have individual-level data on home tap water, our estimates rely on previous studies for data on arsenic in formula powder (Jackson et al. 2012) and bottled water (Sullivan and Leavey 2011). Although we had a fairly small sample of formula-fed babies, the mean formula ingestion rate from our population (0.81 L/day) is consistent with feeding recommendations from the American Academy of Pediatrics, Committee on Practice and Ambulatory Medicine, Bright Futures Periodicity Schedule Workgroup (2014) and the mean body weight–adjusted ingestion rate from our population is identical to the value recommended by the U.S. EPA (2008) for breast milk. Finally, breast milk was collected into the containers typically used by each parent, including plastic bags and bottles. Low levels of arsenic in these containers may have leached into breast milk and thus would overestimate exposure for infants fed milk directly from the breast rather than pumped. However, because 39% of parents reported feeding pumped breast milk, this was considered a conservative assumption, especially because arsenic contamination of plastic has not been reported in the literature.

We expect that population-wide arsenic exposure will increase during the second part of the first year of life, as the prevalence of formula-feeding increases and as solid foods are introduced. For example, rice, rice cereal, and common infant foods containing rice as a thickening agent can contain elevated concentrations of arsenic (Abedin et al. 2002; Hernández-Martínez and Navarro-Blasco 2013; Jackson et al. 2012; Meharg 2004). Also, rice has been shown to contribute to arsenic exposure in older children (Davis et al. 2012) and pregnant women (Gilbert-Diamond et al. 2011).

In conclusion, our findings suggest that breastfed infants have lower exposure to arsenic than formula-fed infants, even when drinking-water arsenic concentrations are low (< 1 μg/L). Moreover, our estimates suggest that both formula powder and drinking water can be sources of arsenic exposure for U.S. infants.

## Supplemental Material

(278 KB) PDF
