# Erratum: Estimated Exposure to Arsenic in Breastfed and Formula-Fed Infants in a United States Cohort

**DOI:** 10.1289/ehp.123-A117

**Published:** 2015-05-01

Carignan CC, Cottingham KL, Jackson BP, Farzan SF, Gandolfi AJ, Punshon T, Folt CL, Karagas MR. 2015. Environ Health Perspect 123:500–506; http://dx.doi.org/10.1289/ehp.1408789

Published online 23 February 2015

The original Advance Publication of this article included the following errors: In the Abstract, the maximum urinary arsenic concentration was initially rounded to 3.0 µg/L and corrected to the value of 2.9 µg/L, and the median daily arsenic intake estimates for formula-fed and breastfed infants were transposed. In the Results section, the proportion of homes in the infant substudy with tap water > MCL was incorrectly reported as 2% instead of 1%. These errors have been corrected. In Figure 1, a few duplicate data points introduced by the software used to generate the figure have been removed.

The authors regret the errors.

