# Supplementary material for: Estimated Exposure to Arsenic in Breastfed and Formula-Fed Infants in a United States Cohort
Source: Environ Health Perspect. 2015 Feb 23;123(5):500–6. doi: 10.1289/ehp.1408789 (PMC4421773; doi:10.1289/ehp.1408789)
Supplement: (278 KB) PDF [file ehp.1408789.s001.508.pdf]

Environmental Health Perspectives doi:10.1289/ehp.1408789

Supplemental Material

Estimated Exposure to Arsenic in Breastfed and Formula-Fed Infants in a United States Cohort

Courtney C. Carignan, Kathryn L. Cottingham, Brian P. Jackson, Shohreh F. Farzan, A. Jay Gandolfi, Tracy Punshon, Carol L. Folt, and Margaret R. Karagas

Table S1. Summary statistics for feeding data for NHBCS infants between 1-3 months of age, based on the two full days of 3-day food diaries completed by the mother (n=115). Includes all diaries returned through September 2013, regardless of urine sample status).

Feeding Mode and Variable	n (%)	Mean	Min	5th %tile	25th %tile	50th %tile	75th %tile	95th %tile	Max
Exclusively formula-fed	15 (13%)								
Formula (L/d)		0.81	0.58	0.58	0.71	0.77	0.89	1.18	1.18
Formula feedings per day		8.0	5.5	5.5	6.5	7.5	9.0	12.0	12.0
Mixed (formula + breast milk)	19 (17%)								
Formula (L/d)		0.30	0.005	0.005	0.06	0.29	0.47	0.75	0.75
Formula feedings per day		3.8	0.5	0.5	1.0	3.0	5.5	9.0	9.0
Breast milk feedings per day		6.6	0.5	0.5	4.5	6.5	8.5	10.5	10.5
Minutes per day on breast		130	0	0	40	135	218	300	300
Exclusively breastfed	81 (70%)								
Breast milk feedings per day		9.5	1.0	6.5	8.0	9.0	10.5	14.5	17.0
Minutes per day on breast		142	0	0	90	134	190	356	403

Table S2. Geometric mean (GM) and maximum urinary arsenic concentrations by feeding mode, without correction for specific gravity. Due to low detection frequencies for the individual arsenic species, only the detection frequencies (as %) and maximum concentrations (μg/L) are presented for those data.

Urinary Arsenic Measurement	LOD[a]	Breastfed (n=48) GM or % (Max)	Mixed (n=13) GM or % (Max)	Formula-fed (n=11) GM or % (Max)	All infants (n=72) GM or % (Max)
Total As	0.05	0.17 (1.45)	0.36 (1.15)	0.81 (3.35)	0.25 (3.35)
UAs[b]	NA	0.09 (1.08)	0.29 (1.15)	0.80 (2.89)	0.16 (2.89)
AsB	0.06	23% (0.97)	15% (0.46)	9% (0.78)	19% (0.97)
Inorganic As[c]	0.17	25% (0.49)	23% (0.46)	18% (0.33)	24% (0.49)
MMA	0.11	0% (<LOD)	23% (0.50)	27% (0.26)	8% (0.50)
DMA	0.15	19% (0.51)	54% (3.23)	100% (1.04)	38% (3.23)

[a]LOD = limit of detection. [b]UAs = Urinary arsenic calculated as total arsenic – AsB. [c]Inorganic As includes As(III) and As(V).

NA: Urinary arsenic calculated as the total arsenic – AsB, so there is no single LOD.

Table S3. Estimated exposure to arsenic via breast milk and formula based on exposure models for exclusively breastfed and exclusively formula-fed infants.

Component of Infant Diet	µg As/d Min	µg As/d Median	µg As/d Max	µg As kg^{-1} d^{-1} Min	µg As kg^{-1} d^{-1} Median	µg As kg^{-1} d^{-1} Max
Breast milk[a]	<LOD	0.25	0.73	<LOD	0.04	0.10
Powdered formula[b] mixed using:						
Tap water from the NHBCS[c]	0.17	1.24	226	0.04	0.22	31
Bottled water[d]	0.21	1.39	4.4	0.05	0.25	0.60
1 µg/L	0.75	1.69	3.31	0.17	0.30	0.45
5 µg/L	3.06	4.92	8.04	0.68	0.88	1.10
10 µg/L	5.94	8.96	14.0	1.32	1.60	1.91

LOD = limit of detection

[a]Breast milk estimates derived from measurements made in this study (Table 2). [b]Arsenic concentrations in powdered formula based on the New Hampshire market-basket study conducted by Jackson et al. (2012). [c]Measurements of home tap water arsenic were made as part of this study (Table 2). [d]Arsenic concentrations in bottled natural spring water based on the California market-basket study conducted by Sullivan and Leavey (2011).

References

Jackson BP, Taylor VF, Punshon T, Cottingham KL. 2012. Arsenic concentration and speciation in infant formulas and first foods. Pure Applied Chem 84:215-224.

Sullivan MJ, Leavey S. 2011. Heavy metals in bottled natural spring water. J Environ Health 73:8-13.

USEPA (U.S. Environmental Protection Agency). 2008. Child-Specific Exposure Factors Handbook (Final Report). EPA Publication no. EPA/600/R-06/096F. Available: http://cfpub.epa.gov/ncea/cfm/recordisplay.cfm?deid=199243 [accessed 10 April 2014].
